# Explainable Artificial Intelligence Unravels the Possible Distinct Roles of VKORC1 and CYP2C9 in Predicting Warfarin Anticoagulation Control

**DOI:** 10.3390/medsci14010156

**Published:** 2026-03-22

**Authors:** Kannan Sridharan, Gowri Sivaramakrishnan

**Affiliations:** 1 Department of Pharmacology & Therapeutics, College of Medicine & Health Sciences, Arabian Gulf University, Manama P.O. Box 26671, Bahrain; 2 Bahrain Defence Force Royal Medical Services, Riffa P.O. Box 28743, Bahrain; gowri.sivaramakrishnan@gmail.com

**Keywords:** warfarin, artificial intelligence, XAI, personalized therapy

## Abstract

**Background:** Warfarin pharmacogenomics is critical due to its narrow therapeutic index and significant interpatient variability. While machine learning (ML) can predict anticoagulation control status (ACS), its “black-box” nature limits clinical translatability. Explainable Artificial Intelligence (XAI) addresses this by providing interpretable insights. This study applied ML and XAI to a warfarin pharmacogenomic dataset to predict poor ACS and explain model decisions. **Methods:** A post hoc analysis was conducted on a cross-sectional dataset of 232 patients receiving warfarin for ≥6 months. Data included age, gender, interacting drugs, SAMe-TT2R2 score, and genotypes for CYP2C9, VKORC1, and CYP4F2. Poor ACS was defined as time in therapeutic range (TTR) < 70%. The dataset was split into training (70%) and testing (30%) cohorts. Three models, Random Forest, XGBoost, and Logistic Regression, were developed and evaluated using AUC-ROC, sensitivity, and specificity. XAI techniques, including permutation importance and SHapley Additive exPlanations (SHAP), were employed for global and local interpretability. **Results:** Of 232 patients, 141 (60.8%) had poor ACS. XGBoost and Random Forest demonstrated comparable predictive accuracy (AUC-ROC: 0.67), outperforming Logistic Regression. Sensitivity was 0.83 and 0.79 for XGBoost and Random Forest, respectively. However, specificity was modest for both ensemble methods (Random Forest: 0.48; XGBoost: 0.41) and extremely low for Logistic Regression (0.04), indicating poor discrimination, particularly for identifying patients with adequate anticoagulation control. Globally, important predictors included the age, SAMe-TT2R2 score, CYP2C9 (*2/*2), female gender, and VKORC1 (C/T). XAI revealed predictions were primarily driven by VKORC1, CYP4F2, SAMe-TT2R2 scores, and drug interactions. Concordance between XAI predictions and actual ACS was 78% for adequate and 88.6% for poor ACS. SHAP analysis showed VKORC1 provided a stable risk signal (mean absolute SHAP: 1.44 ± 0.49 in concordant cases), while CYP2C9 was a high-variance, high-impact driver of discordance (mean SHAP: 3.44 ± 3.79 in discordant cases). **Conclusions:** ML models, particularly ensemble methods, show modest ability to predict poor warfarin control with limited ability to correctly identify patients with adequate control from our dataset. XAI transforms these models into interpretable tools, with SHAP analysis attributing predictions to specific genetic and clinical features. While predictive accuracy remains modest, this approach enhances transparency and provides a foundation for generating hypotheses that may ultimately support clinical decision-making in pharmacogenomic-guided warfarin therapy.

## 1. Introduction

Warfarin remains a commonly used oral anticoagulant for the prevention and treatment of thrombotic disorders. Although its clinical use has declined in recent years with the advent of direct oral anticoagulants, studies still report its use in approximately 42.2% of cases [1]. Warfarin’s narrow therapeutic index, coupled with its numerous drug–drug and drug-food interactions, poses significant clinical challenges. Maintaining patients on an appropriate dosing regimen requires regular monitoring of its therapeutic effect via the prothrombin time international normalized ratio (PT-INR) [2]. A Norwegian study found that within one year of initiation, 16% of patients switched from warfarin to another oral anticoagulant [3]. Furthermore, research in long-term care settings revealed the prevalence of warfarin use ranging from 17% to 57%, with warfarin-related adverse events reported at a rate of 25.5 per 100 resident-months of therapy [4].

The genetic determinants of warfarin’s therapeutic response and adverse events are well-established. Critical roles have been identified for the warfarin-metabolizing enzyme Cytochrome P450 2C9 (CYP2C9), the vitamin K-metabolizing enzyme CYP4F2, and warfarin’s pharmacodynamic target, vitamin K epoxide reductase complex 1 (VKORC1) [5]. A recent meta-analysis of genome-wide studies confirmed that polymorphisms in CYP2C9 and VKORC1 are the most important in determining stable warfarin dose [6]. Reflecting this evidence, the Clinical Pharmacogenetics Implementation Consortium (CPIC) guideline recommends genotyping for CYP2C9, VKORC1, and CYP4F2 to personalize therapy and achieve target INR [7].

Machine learning (ML) algorithms are sophisticated statistical tools capable of identifying complex, hidden relationships between variables to predict outcomes, holding considerable potential for personalized medicine. Given the multifactorial nature of warfarin response, involving both genetic and non-genetic predictors, various ML algorithms have been developed and shown to successfully identify these predictive factors. In previous work, we applied classification, regression algorithms, and deep neural networks to identify predictors of adequate anticoagulation control status (ACS) with warfarin [8,9]. However, a key limitation of these studies was their “black-box” nature, which obscured the reasoning behind individual predictions of poor ACS. We were unable to elucidate how predictor variables interacted for specific cases or to analyze the factors distinguishing concordant from discordant predictions (i.e., matches or mismatches between predicted and actual ACS).

Recently, Explainable Artificial Intelligence (XAI) has emerged as an advancement in the AI field, providing tools to generate human-understandable explanations for AI decisions without compromising predictive accuracy [10]. Integrating XAI into model-based research shifts the focus from merely reporting performance metrics to explaining the decision-making process. This makes findings more interpretable, trustworthy, and directly applicable to personalized clinical decision-making. Therefore, the present study was conducted to incorporate XAI into machine learning analyses of a warfarin pharmacogenomic dataset.

## 2. Methods

### 2.1. Study Design and Ethics

This investigation was conducted as a post hoc analysis of data originally gathered for a warfarin pharmacogenomics research project, a cross-sectional study [11]. The protocol for the original research received approval from the Institutional Ethics Committee (reference E024-PI-11/18). Data collection occurred from September 2019 to November 2020. All participants provided written informed consent voluntarily, and the study adhered to the principles outlined in the most recent version of the Declaration of Helsinki.

### 2.2. Study Procedure

Full methodological details regarding patient recruitment have been published previously [8]. The present study focuses specifically on the application of machine learning and XAI to this dataset; therefore, to maintain focus on the novel analytical approach and avoid redundancy, those detailed statistical comparisons are not reproduced herein. Eligibility for inclusion required that patients prescribed warfarin as standard therapy for a minimum duration of six months and attended an anticoagulation clinic for PT-INR monitoring. For each participant, the following data were extracted: age, gender, PT-INR values, and concurrent medications. The SAMe-TT2R2 score, which incorporates sex, age, medical history, treatment, tobacco use, and race, was calculated for every individual [12]. Concomitant medications classified as interacting with warfarin included statins, proton pump inhibitors, carbamazepine, phenytoin, valproic acid, and amiodarone [8]. Genotyping for specific polymorphisms in *CYP2C9* (rs1799853 and rs1057910), *VKORC1* (rs9923231), and *CYP4F2* (rs2108622) were performed using established allele discrimination techniques, as detailed in prior work [8]. The target therapeutic PT-INR range was defined as 2.5–3.5 for patients who had undergone mechanical heart valve replacement surgery. For all other clinical indications, the target range was 2–3 [13]. TTR was estimated for each patient using the linear interpolation method developed by Rosendaal [14]. Based on this TTR, ACS was categorized; a TTR exceeding 70% denoted adequate ACS, while a TTR below 70% indicated poor ACS [15].

### 2.3. ML and XAI Analyses

Continuous variables were expressed as mean ± standard deviation, while categorical variables were presented as frequencies and percentages for the exploratory data analysis. Categorical variables including CYP2C9 genotypes (*1/*1, *1/*2, *1/*3, *2/*2, *2/*3, and *3/*3), CYP4F2 genotypes (C/C, C/T, and T/T), VKORC1 genotypes (C/C, C/T, and T/T), CYP2C9 metabolizer status (poor, intermediate and normal), gender (male and female), interacting drug status (present and absent), and SAMe-TT2R2 scores (0–3) were converted to factors. Although the SAMe-TT2R2 score represents an ordinal measure, it was treated as categorical to capture potential non-linear relationships with anticoagulation outcomes. Data preprocessing was conducted with careful attention to avoid data leakage. Specifically, the dataset was first partitioned into training (70%) and testing (30%) subsets using stratified random sampling. All subsequent preprocessing steps were performed by fitting transformations exclusively on the training data and then applying these fitted transformations to the test data. For continuous variables, z-score standardization was performed using the mean and standard deviation calculated from the training set; these same parameters were then applied to standardize the test set. For categorical variables, one-hot encoding was performed by first identifying all category levels present in the training data and creating dummy variables based on these levels. The test data were then encoded using the same dummy variable structure, with any categories appearing only in the test set (none occurred in this dataset) being handled appropriately. This approach ensures that no information from the test set influences the preprocessing transformations, thereby preserving the integrity of the train-test split and providing unbiased estimates of model performance on unseen data.

In the training set, the distribution of ACS was 64 patients (39.3%) with adequate control and 99 patients (60.7%) with poor control. In the testing set, the distribution was 27 patients (39.1%) with adequate control and 42 patients (60.9%) with poor control, confirming successful stratification. Statistical comparison between training and testing cohorts were carried out using Chi-square/Fisher’s exact probability tests for categorical variables and Wilcoxon-signed rank sum test for numerical variables. Class imbalance was addressed through balanced sampling strategies and appropriate weighting during model training. Specifically, for the Random Forest model, we employed the following strategy: the sampsize parameter was set to the size of the minority class (adequate control) to ensure balanced samples, and the nodesize parameter was reduced to 1 to allow trees to grow deeply enough to capture minority class patterns. For the XGBoost model, we implemented scale_pos_weight, calculated as the ratio of negative to positive class instances (approximately 0.65:1 in the training set), which assigns higher penalties to misclassifications of the minority class. For Logistic Regression, we applied case weights inversely proportional to class frequencies during model fitting. These techniques were applied only to the training data, with the testing set remaining untouched to preserve an unbiased evaluation of model performance on the original class distribution. Regarding hyperparameter optimization, we acknowledge that a systematic tuning approach was not employed in this study. For Random Forest, the number of trees was set to 500 and the mtry parameter (number of predictors considered at each split) was set to the square root of the total number of predictors, following default recommendations in the R package. For XGBoost, we used 100 boosting iterations, a maximum tree depth of 6, a learning rate (eta) of 0.1, and L2 regularization (lambda) with default values. These parameters were selected based on commonly used values in the literature rather than through systematic optimization techniques such as grid search, cross-validation, or early stopping. We recognize that the absence of formal hyperparameter tuning, including k-fold cross-validation to guide parameter selection and early stopping to prevent overfitting in XGBoost, represents a significant methodological limitation. This may have resulted in suboptimal model performance and increases the risk of overfitting or underfitting. Future work should incorporate rigorous hyperparameter optimization using techniques such as grid search or random search with cross-validation to identify optimal model configurations and enhance predictive performance.

Model performance was evaluated using comprehensive metrics including accuracy, area under the receiver operating characteristic curve (AUC-ROC), sensitivity, specificity, precision, F1-score, Cohen’s kappa, and balanced accuracy along with calibration statistics. Model calibration was evaluated using multiple quantitative metrics in addition to visual calibration plots. These included the Brier score (mean squared difference between predicted probabilities and actual outcomes, with lower values indicating better calibration), log loss (cross-entropy loss, with lower values indicating better performance), and the calibration slope (slope of the regression of observed outcomes on predicted probabilities, with values closer to 1 indicating ideal calibration). These metrics provide a more rigorous basis for comparing the calibration performance of the three modeling approaches. XAI techniques were employed to enhance interpretability of model predictions. Global interpretability was achieved through permutation feature importance calculated by random permutation of feature values and measurement of AUC degradation. Local interpretability was facilitated through identification of individual feature contributions for predicting poor ACS for each of the included patients. SHAP (SHapley Additive exPlanations) values were calculated to interpret the contribution of each feature to individual model predictions. SHAP values represent the marginal contribution of a feature to the model’s output relative to a baseline expectation. A positive SHAP value indicates that the feature increased the predicted probability of the positive class (poor anticoagulation status), while a negative SHAP value indicates that the feature decreased this probability, favoring the negative class (adequate status). The magnitude of the SHAP value reflects the strength of the feature’s influence. For group-level interpretation, we computed mean SHAP (the average SHAP value across a subgroup), which indicates the net directional influence of a feature, whether it tends to push predictions toward poor or adequate outcomes on average. Additionally, mean absolute SHAP was calculated as the average of absolute SHAP values, representing the overall magnitude of influence irrespective of direction, thereby quantifying the average impact strength of each feature across predictions. These metrics allowed us to compare feature importance and influence patterns between concordant and discordant prediction groups. All analyses were carried out using R software (version 4.5.1).

## 3. Results

### 3.1. Exploratory Data Analysis

**Figure 1:**
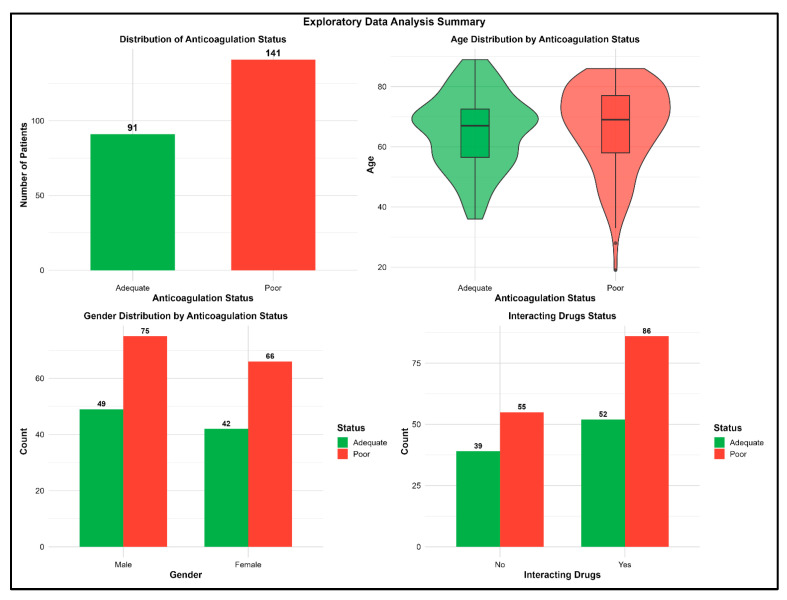
Plots of Exploratory Data Analysis with non-genetic variables.

**Figure 2:**
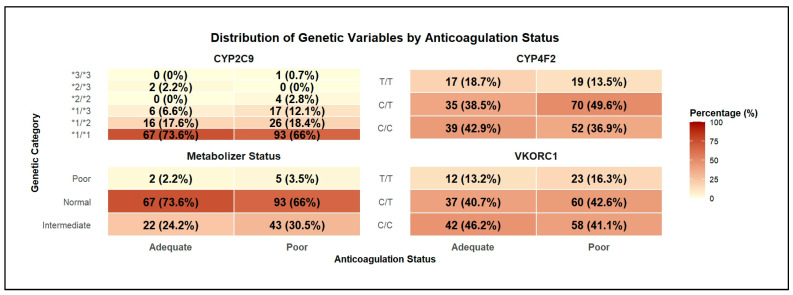
Genetic Profile of Patients with Anticoagulation Status.

A total of 232 patients’ data were included of which 141 (60.8%) had poor ACS. Similar distributions of age, gender and proportion of interacting drugs were observed between those with poor and adequate ACS (Figure 1). Genotype distributions revealed that all with CYP2C9 (*2/*2 and *3/*3) had poor ACS (Figure 2). It is important to note that these genotype categories represent small subgroups within the cohort. While the observation that all patients in these subgroups had poor ACS is descriptively accurate, the small cell counts mean that this finding should be interpreted with caution. Such small subgroups can create instability in model estimation, increase the risk of overfitting when these variables are included as predictors, and may lead to perfect separation issues in regression-based approaches. This consideration is particularly relevant for the Logistic Regression model, where these small subgroups may have contributed to the extreme coefficient estimates and poor specificity observed. In the ensemble methods, while tree-based algorithms can handle such sparse categories, the instability remains a limitation for reliable inference from these specific genotype combinations.

### 3.2. Random Forest, XGBoost and Logistic Regression Analyses

Training and testing cohorts had similar distributions of predictor variables (Table 1).

**Figure 3:**
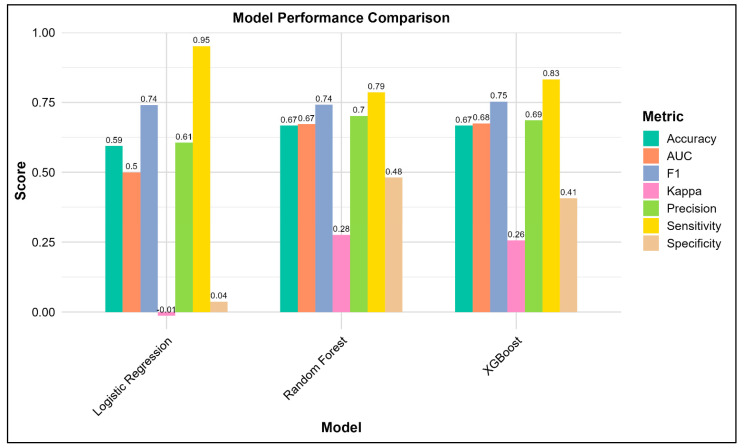
Comparison of Model Performances in Predicting Poor ACS.

**Figure 4:**
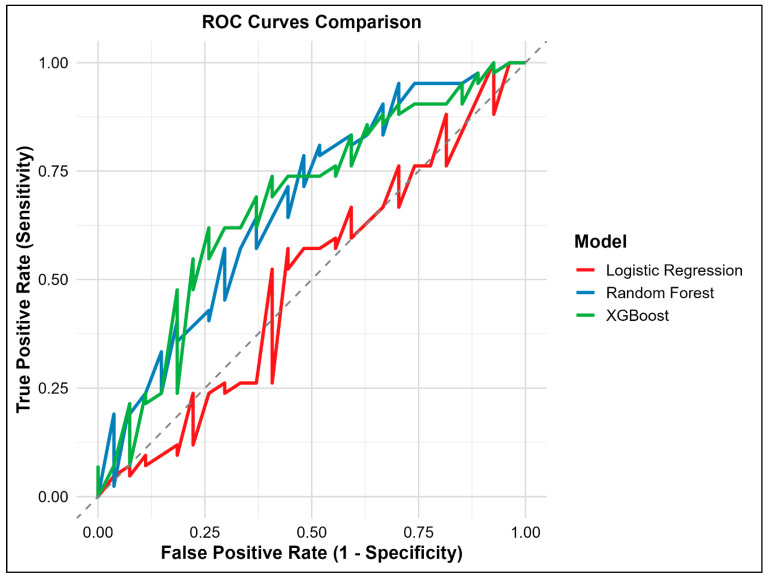
ROC for Model Accuracies in Predicting Poor ACS.

**Figure 5:**
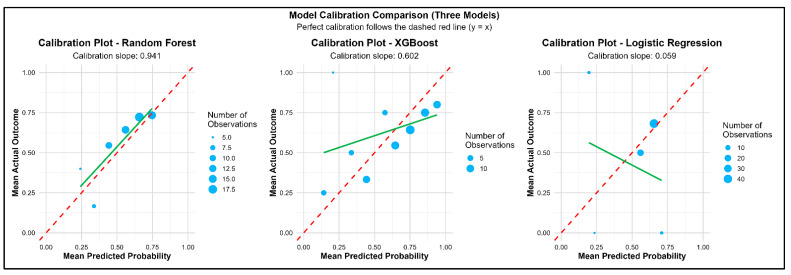
Calibration Plots of Predicted Probability of Poor ACS across Models.

**Figure 6:**
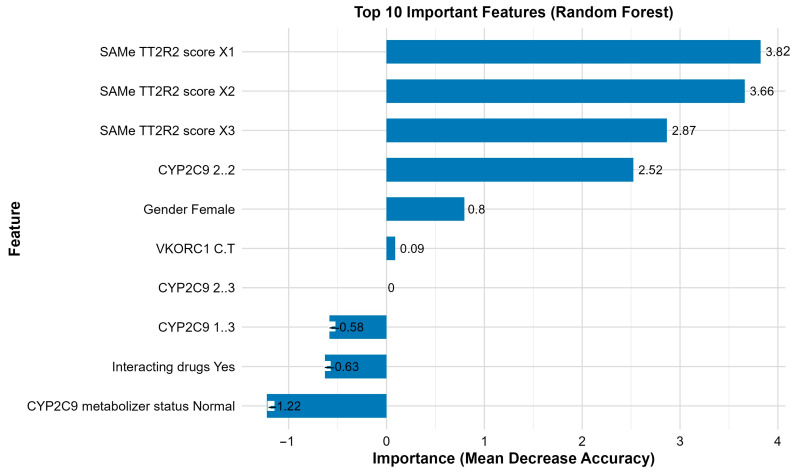
Top Features of Importance as Predicted by Random Forest Model.

Of the three models, XGBoost and Random Forest had similar accuracies (AUC-ROC: 0.67) with a sensitivity of 0.83 and 0.79, respectively, in predicting poor ACS (Figure 3). Both XGBoost and Random Forest were observed with significant predictive accuracies rather than Logistic Regression (Figure 4). Table 2 lists all key evaluation metrics for all three models in testing cohort that also corroborate the inferior predictive ability of Logistic Regression model. However, examination of specificity reveals important limitations. Random Forest achieved a specificity of only 0.48, correctly identifying just 13 of 27 patients with adequate anticoagulation control, while XGBoost achieved a specificity of 0.41 (11 of 27 patients). The Logistic Regression model performed particularly poorly, with a specificity of 0.04, correctly identifying only 1 of 27 patients with adequate control. These specificity values indicate that all models, particularly Logistic Regression, have limited ability to discriminate patients with adequate anticoagulation control, resulting in a high rate of false positives. The balanced accuracy scores further reflect this modest overall discriminative performance. The extremely low specificity of the Logistic Regression model, despite the application of weighting for class imbalance, indicates that the imbalance handling was inadequate. This finding underscores the vulnerability of simpler models to class imbalance and the relative robustness of ensemble methods in this context. Calibration plots revealed Random Forest to be the best predictor model for the study population (Figure 5). SAMe-TT2R2 score followed by CYP2C9 (*2/*2) genotype, female gender and VKORC1 (C/T) genotype were identified to be the top features of importance in predicting poor ACS (Figure 6).

### 3.3. XAI Analysis

**Figure 7:**
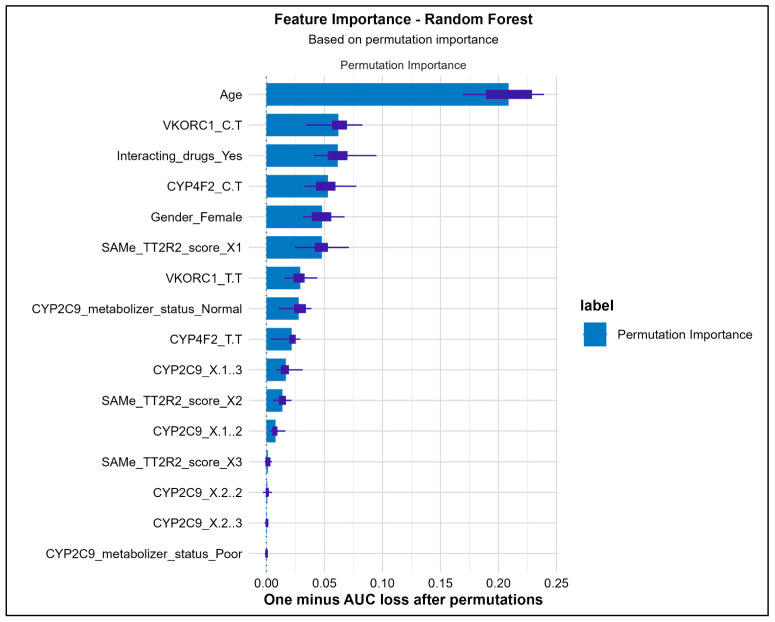
Variables of Importance Identified by Random Permutation of Feature Values.

**Figure 8:**
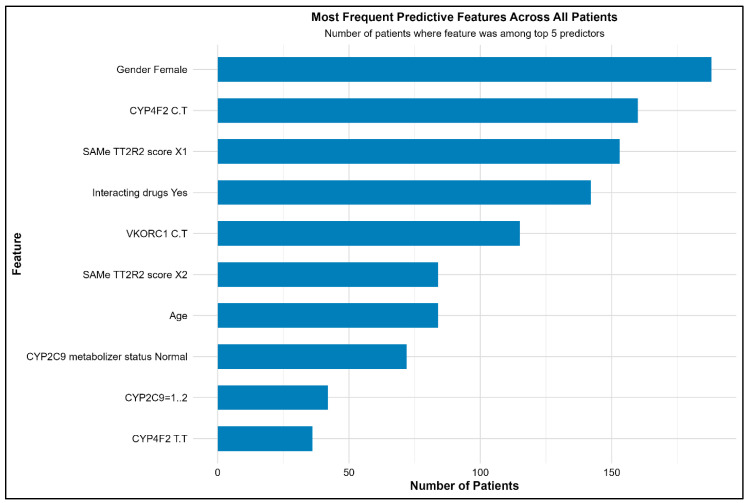
Top Features Identified by the XAI Explaining the Poor ACS.

Figure 7 lists the variables of importance based on the permutation method that has identified age, VKORC1 (C/T), presence of interacting drugs, CYP4F2 (C/T), and female were the top 5 features predictive of poor ACS amongst the entire cohort of patients as identified globally. The XAI analysis revealed that genetic variants in VKORC1 and CYP4F2, along with clinical factors such as gender and drug interactions, were the primary drivers of predictions for poor ACS. It is important to note that these attributions reflect the model’s decision-making process rather than establishing direct biological causation. Age and gender contributed moderately but consistently across patients. SHAP values indicated both risk-enhancing and protective influences as learned by the model from the training data, providing transparent insight into individual-level predictions and potential misclassifications. The local interpretability data revealed female gender (n = 188, 81.03%), CYP4F2 (C/T) genotype (n = 160, 69%), SAMe-TT2R2 score (n = 153, 65.9%), presence of interacting drugs (n = 142, 61.2%) and VKORC1 (C/T) genotype (n = 115, 49.6%) along with others (Figure 8).

The XAI prediction results were concordant in 78% of those with adequate ACS and with a slightly higher rate (88.6%) amongst those with poor ACS. To understand why the model succeeded or failed in individual cases, we stratified SHAP values by concordance status (Table 3). It is important to note that these stratified analyses are based on relatively small subgroups, particularly the discordant group (n = 9 for CYP2C9 variants). Consequently, the mean SHAP values and standard deviations reported for these subgroups should be interpreted with caution, as they may be unstable and sensitive to individual patient characteristics. No statistical testing was performed to compare SHAP values between concordant and discordant groups, as the small sample sizes would render such tests underpowered and potentially misleading. The following observations are therefore presented as exploratory and hypothesis-generating, providing preliminary insights that require validation in larger cohorts.

In concordant cases, genetic features demonstrated strong and consistent influences from the model. VKORC1 variants exhibited the highest mean absolute SHAP value (1.44 ± 0.49) with a clear directional bias (88.8% positive), indicating a stable risk-enhancing effect as captured by the model. CYP2C9 variants showed the largest magnitude of influence (mean SHAP values = 2.31 ± 1.61) but with more balanced directional distribution. Clinical features such as SAMe-TT2R2 scores (mean SHAP values = 1.21 ± 0.72) and drug interactions (mean SHAP values = 1.08 ± 0.20) also contributed substantially.

In discordant cases, a different pattern emerged. CYP2C9 exhibited extreme variability (mean SHAP values = 3.44 ± 3.79), reflecting high impact but conflicting genetic contributions. However, given the small sample size (n = 9) driving this estimate, this finding should be regarded as tentative and requiring confirmation in larger datasets. While VKORC1 maintained a strong risk direction (80% positive), its absolute impact was attenuated compared to concordant cases (1.26 ± 0.40 vs. 1.44 ± 0.49). Demographic features such as age and gender showed reduced directional consistency, with age shifting toward a net protective effect (mean SHAP = −0.32) compared to a slight risk effect in concordant cases (mean SHAP = 0.09).

These exploratory findings suggest, as a hypothesis for future investigation, that discordant predictions may arise not from the absence of influential features, but from conflicting directional signals and higher variability within key predictors, particularly CYP2C9. From a model attribution perspective, this generates the hypothesis that CYP2C9 variants may act as a primary source of predictive complexity, while VKORC1 provides a more stable, predictable baseline signal in the model’s decision framework. Rigorous validation in larger, independent cohorts is essential to confirm these preliminary observations.

**Figure 9:**
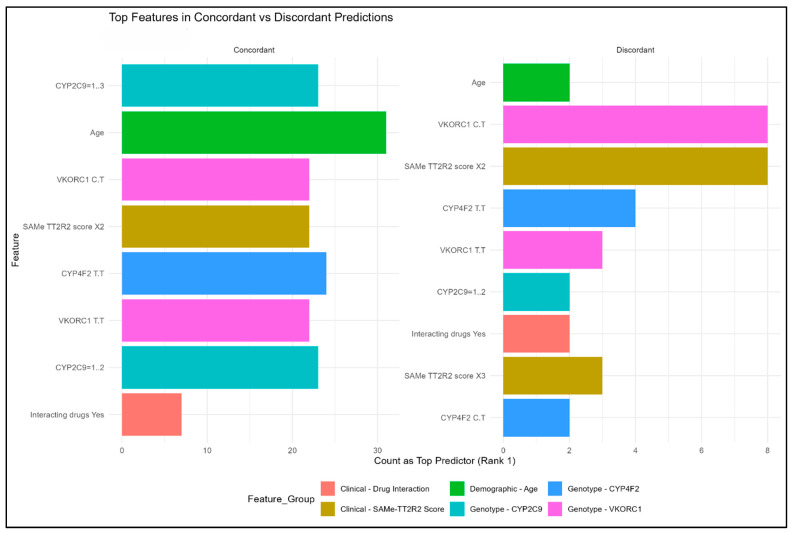
Top Features in Concordant and Discordant Predicted Groups.

**Figure 10:**
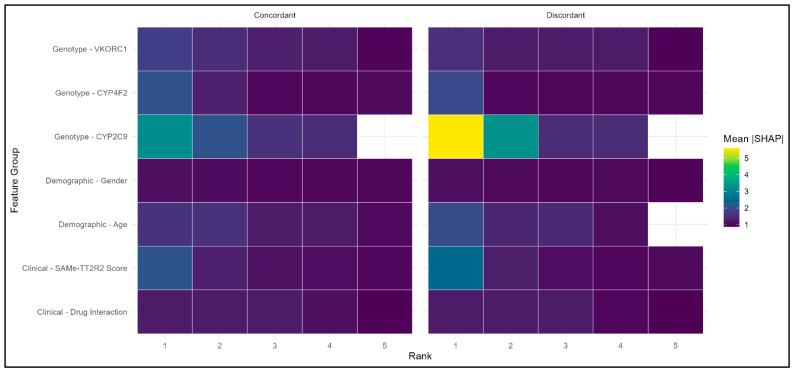
Heatmap Analysis of Feature Importance Between Concordant and Discordant Groups.

The analysis of top predictive factors amongst the concordant group revealed the following ranking of variables: age, CYP4F2 (T/T) genotype, CYP2C9 *1/*2 and *1/*3 genotypes, VKORC1 C/T and T/T genotypes, and SAMe-TT2R2 scores (Figure 9). However, comparison with the discordant group (Figure 10) highlights the distinctive roles of key pharmacogenes: VKORC1 maintains stable, predictable influence across both groups, whereas CYP2C9 emerges as a high-variance driver whose importance surges in discordant cases. This disparity implies that while VKORC1 provides a predictable baseline, CYP2C9 variants act as the critical differentiator that complicates clinical modeling and drives the most significant discrepancies in predictive accuracy.

## 4. Discussion

### 4.1. Statement of Key Findings

This study applied and compared ML and XAI approaches to a warfarin pharmacogenomic dataset to predict poor anticoagulation control. While ensemble methods (Random Forest and XGBoost) demonstrated moderate and superior predictive accuracy compared to logistic regression, the XAI analysis provided critical, nuanced insights into the model’s decision-making. The SAMe-TT2R2 score and specific genotypes (CYP2C9 *2/*2 and VKORC1 C/T) were identified as top global predictors. However, SHAP-based interpretation revealed that predictions were primarily driven by a complex interplay of genetic (VKORC1, CYP4F2, and CYP2C9) and clinical factors (drug interactions, SAMe-TT2R2 score), with age and gender providing consistent secondary contributions as captured by the model. Notably, the analysis uncovered a fundamental distinction in how the model utilizes these features: VKORC1 acted as a stable, baseline risk factor in model predictions, whereas high-variance CYP2C9 genotypes emerged as the critical differentiator, with their conflicting directional influences representing a major source of model discordance and predictive complexity. These observations, derived from exploratory subgroup analyses with small sample sizes, should be interpreted as hypothesis-generating rather than definitive. They provide directions for future investigation rather than established mechanistic conclusions.

### 4.2. Comparison with Existing Literature

Our ML performance was observed to be like the previous studies where they outperformed Logistic Regression analysis [8,9]. Although, both Random Forest and XGBoost were identified with similar accuracies, the calibration plots revealed superiority of Random Forest over XGBoost. However, the modest AUC values (0.67) and limited specificities (0.41–0.48) observed in our study underscore that predictive performance remains a significant challenge in this domain. The ensemble methods, while superior to logistic regression, still misclassify approximately half of patients with adequate anticoagulation control, limiting their potential clinical utility. Random Forest improves model stability by combining the predictions from several decision trees while XGBoost applies gradient boosting technique and is preferred for unbalanced datasets [16]. The predictive accuracies of both these ensemble models have been observed to outperform the traditional classification and decision tree algorithms [8]. Nevertheless, the modest discriminative performance emphasizes the critical requirement of XAI not as a supplement to strong prediction, but as a tool for understanding the features contributing to model decisions and for generating hypotheses about the factors that complicate accurate prediction. However, the predictive accuracies of both the ensemble models used in this study were moderate, thus emphasizing the critical requirement of XAI for understanding the features contributing to model prediction for a better clinical translation. XAI analysis revealed that both genotypes of VKORC1 (C/T) and CYP4F2 (C/T) along with non-genetic factors (interacting drugs and female gender) were the top variables predicting poor ACS. However, a pivotal discovery was the distinct roles these genetic features played in the model’s predictions. VKORC1 exhibited a strong, stable, and consistently risk-enhancing influence, particularly in concordant predictions (mean absolute SHAP: 1.44 ± 0.49; 88.8% positive). This suggests its effect as learned by the model is a reliable, baseline signal for increased warfarin sensitivity and difficulty in achieving stable control, which is consistent with its well-established role in warfarin’s pharmacodynamics [17]. Contrastingly, CYP2C9 variants displayed the largest magnitude of influence but with high variance and less directional consistency in model attributions possibly attributed to a very few numbers of individuals with these variants. This was dramatically amplified in discordant predictions, where its mean SHAP value surged (3.44 ± 3.79) and exhibited extreme variability. However, given that this estimate is derived from only 9 discordant cases, this finding must be considered preliminary and hypothesis-generating. It generates the hypothesis that CYP2C9 may serve as a primary source of predictive ambiguity, but this requires confirmation in larger studies with adequate statistical power for subgroup analyses. This distinction, elegantly revealed by XAI, suggests potential separate clinical contributions of these genes that warrant further investigation. Furthermore, the stratified SHAP analysis by concordance status provided a novel diagnostic lens into model failures. It demonstrated that discordant predictions did not result from a lack of influential features but from conflicting directional signals and heightened variability within those features. For instance, in discordant cases, the protective influence of interacting drugs was less consistent, and the directional effect of age shifted. This indicates that for certain patients, the model receives ambiguous or contradictory evidence from the input features, leading to misclassification. This insight is invaluable for model refinement and clinical caution, suggesting that patients with specific combinations of genetic variants (particularly certain CYP2C9 genotypes) and clinical factors may inherently reside in a “grey zone” of predictive uncertainty. The high local interpretability rate, where key features like female gender and CYP4F2 (C/T) genotype were identifiable for over two-thirds of individual predictions, demonstrates the practical feasibility of using XAI to generate patient-specific explanations. This transforms the model from an opaque predictor into a tool that can, for each patient, highlight the top clinical and genetic factors contributing to their estimated risk. Such transparency is a prerequisite for building clinician trust and forms the basis for actionable, personalized insights rather than a standalone risk score.

### 4.3. Strengths, Limitations and Way Forward

A key strength of this study is the integration of XAI with established ML algorithms, moving beyond traditional “black-box” predictive modeling. By employing SHAP, we have not only identified global feature importance, confirming known clinical and genetic factors like the SAMe-TT2R2 score and VKORC1 variants, but also provided unprecedented granularity into individual-level predictions. Crucially, we emphasize that these SHAP-based insights represent model attributions, they describe how the model weighs and combines features to arrive at predictions, not direct biological mechanisms. Furthermore, the stratified SHAP analyses comparing concordant and discordant predictions are based on small subgroups (particularly the discordant group, n = 9 for CYP2C9), and no statistical testing was performed to validate observed differences. These analyses are therefore exploratory and hypothesis-generating, providing preliminary insights that require rigorous validation in larger, adequately powered cohorts before any definitive conclusions can be drawn. This dual-level interpretation revealed the distinct roles of key genes: VKORC1 acted as a stable, consistent risk factor, while CYP2C9 emerged as a high-variance, high-impact driver of model discordance. Furthermore, the stratification of SHAP analysis by concordance status offers a novel methodological framework for diagnosing why models fail in specific cases, highlighting that misclassifications stem from conflicting directional influences of features rather than their absence. However, this study has several limitations. Foremost among these is the modest predictive performance of all models, with AUC values of 0.67–0.68 and specificities ranging from 0.41 to 0.48 for the ensemble methods. This indicates that the models have limited ability to discriminate between patients with adequate versus poor anticoagulation control, particularly in correctly identifying those with adequate control. The high false-positive rate would limit clinical utility, as many patients who are well-controlled would be flagged for unnecessary additional monitoring or interventions. First, regarding the temporal nature of our data, it is important to clarify that TTR was estimated using the Rosendaal linear interpolation method, which leverages longitudinal INR measurements to calculate the percentage of time spent within the therapeutic range over each patient’s follow-up period [18]. This approach captures dynamic changes in INR control over time. However, despite this longitudinal outcome ascertainment, our predictive modeling approach treated TTR as a static binary classification (adequate vs. poor control) based on the entire follow-up period, rather than modeling the temporal trajectory of INR control as a dynamic process that could be predicted at multiple time points. Future studies could employ time-varying models or survival analysis approaches to better capture the longitudinal nature of anticoagulation control. Secondly, the sample size represents a significant constraint. This sample is relatively small for tree-based ensemble methods like Random Forest and XGBoost, which typically benefit from larger datasets to fully capture complex patterns without overfitting. The limitation is compounded by our use of one-hot encoding for multiple genotype categories (CYP2C9 with six categories, VKORC1 and CYP4F2 with three categories each), which increases the dimensionality of the feature space and raises the risk of model overfitting, particularly in the test set. Additionally, the stratified subgroup analyses comparing concordant and discordant predictions (with even smaller effective sample sizes within each stratum) should be interpreted with appropriate caution, as these estimates may be unstable. The moderate predictive performance observed in our models likely reflects, at least in part, these sample size and design constraints. Our dataset did not include variables such as dietary vitamin K intake, precise medication adherence metrics, or additional genetic polymorphisms, which could improve model accuracy. Also, the cross-sectional, single-center design of the source study restricts the temporal assessment of ACS and may introduce population-specific biases. Most critically, k-fold cross-validation, bootstrap validation, or external validation on an independent, multi-center cohort was not performed. This represents a substantial limitation, as the absence of external validation leaves the generalizability of our models across different populations, clinical settings, and geographic regions uncertain. Our models should therefore be regarded as hypothesis-generating tools that demonstrate a methodological approach, rather than as clinically ready predictive instruments. Furthermore, as noted in the Section 2, systematic hyperparameter tuning was not performed for any of the machine learning algorithms. The parameters for Random Forest and XGBoost were selected based on literature-derived default values rather than through optimization techniques such as grid search with cross-validation. This increases the risk that our models are not optimally configured for this specific dataset, potentially leading to suboptimal performance or overfitting. The absence of early stopping in XGBoost training further compounds this concern, as it may have allowed the model to continue learning beyond the point of optimal generalization. The predictive performance and XAI-derived insights reported herein may reflect population-specific patterns that could differ in other cohorts. Regarding class imbalance handling, while we employed appropriate techniques including balanced sampling for Random Forest, scale_pos_weight for XGBoost, and inverse probability weighting for Logistic Regression, we acknowledge that a systematic comparison of model performance with and without these balancing techniques was not conducted. The extremely low specificity achieved by the Logistic Regression model, despite weighting, indicates that the imbalance handling was inadequate for this simpler modeling approach. This highlights an important limitation: the effectiveness of imbalance correction techniques varies substantially across model types, and our chosen approach for Logistic Regression proved insufficient to overcome the class imbalance in this dataset. This finding also underscores the relative robustness of tree-based ensemble methods to class imbalance when appropriately configured. Such a comparison would have provided additional insights into the specific impact of imbalance correction on predictive accuracy and is an important consideration for future methodologically focused studies. Most notably, the moderate predictive performance observed in our models indicates that they are not yet suitable for direct clinical implementation. Instead, these findings should be viewed as proof-of-concept for the value of XAI in generating mechanistic insights. Furthermore, our outcome measure was limited to anticoagulation control status (TTR < 70%) and did not encompass clinically important adverse events such as bleeding complications or hypersensitivity reactions. While these outcomes are critical to warfarin safety and patient management, they were not captured in the original dataset and therefore fall outside the scope of the present analysis. However, for clinicians, these findings underscore the critical yet distinct roles of VKORC1 and CYP2C9 genotyping. While VKORC1 provides a reliable baseline for warfarin sensitivity, CYP2C9 status should be recognized as a major source of dosing uncertainty and a potential flag for patients requiring exceptionally vigilant monitoring. The local interpretability offered by XAI tools could, in the future, support clinical decision-making by visualizing the specific factors contributing to an individual’s predicted risk of poor control. For researchers, future work should focus on validating these XAI-derived insights in larger, multi-ethnic, and prospective cohorts. Longitudinal study designs that enable dynamic prediction of INR trajectories at multiple time points would represent a methodological advance over the static classification approach used herein. Such models could better capture the time-varying nature of anticoagulation control and may offer greater clinical utility for guiding monitoring frequency. Sample size calculations should guide future data collection to ensure adequate power for tree-based algorithms and for stratified analyses of interest. Importantly, such studies should also incorporate comprehensive safety outcomes, including bleeding events and other adverse drug reactions, to enable prediction of both efficacy and safety endpoints. The inclusion of these clinically critical outcomes would substantially enhance the translational value of future predictive models. External validation using independent, geographically diverse datasets is an essential prerequisite before any consideration of clinical translation. Additionally, future studies should incorporate systematic hyperparameter optimization using techniques such as grid search or Bayesian optimization with cross-validation to ensure that models are appropriately configured for the data. For boosting algorithms, early stopping based on validation set performance should be employed to mitigate overfitting. In particular, the small subgroup sizes for rare genotypes such as CYP2C9 warrant caution. While the observation that all patients in these subgroups had poor ACS is descriptively accurate, these small counts mean that the model’s handling of these categories is inherently unstable, and any conclusions about the predictive importance of these specific genotypes must be considered tentative. This represents a form of overfitting risk, as the model may assign undue weight to patterns observed in very few patients that may not generalize to larger populations. Such validation studies should assess not only the reproducibility of predictive performance but also whether the distinct mechanistic roles identified for VKORC1 and CYP2C9, specifically, VKORC1 as a stable risk factor and CYP2C9 as a high-variance driver of discordance, hold true across different populations. Incorporating a broader array of clinical (such as diet, and medication adherence), biochemical and genetic variables could enhance model performance. Achieving higher predictive accuracy remains a prerequisite for clinical translation; the current findings provide a methodological framework and highlight key genetic targets for model refinement. The methodological approach of analyzing SHAP value patterns in discordant predictions is a promising strategy for error analysis and model refinement in other pharmacogenomic applications. Ultimately, developing real-time, XAI-integrated clinical decision support systems that provide both a prediction and a transparent rationale represents a vital next step in translating complex ML models into trustworthy tools for personalized warfarin therapy.

Considerations for practical implementation of XAI-supported tools in anticoagulation management merit discussion. However, given the modest predictive performance and limited specificity demonstrated in this study, any discussion of clinical implementation must be preceded by substantial improvements in model accuracy [19]. The current models are not suitable for direct clinical application. In a real-world clinical setting, an XAI-enhanced decision support tool could be integrated into existing electronic health record systems or anticoagulation clinic workflows [20]. Upon entry of a patient’s clinical data (age, gender, interacting medications, SAMe-TT2R2 score) and pharmacogenetic information (CYP2C9, VKORC1, CYP4F2 genotypes), the model generates a risk prediction for poor anticoagulation control. Critically, the XAI component would simultaneously provide a transparent, patient-specific explanation, for example, a visual display showing that the prediction is primarily driven by the patient’s VKORC1 C/T genotype (contributing X% to the risk estimate), the presence of amiodarone therapy (contributing Y%), and an intermediate CYP2C9 metabolizer status (contributing Z%). This explanation could be presented as a simple bar chart or force plot, enabling the clinician to understand not only the risk level but also the specific factors underlying that assessment. Such transparency could support clinical judgment in several ways: by flagging patients who may require more frequent INR monitoring or earlier follow-up appointments; by informing discussions with patients about modifiable factors (such as medication adherence or potential drug interactions); and by building clinician trust in the tool’s recommendations over time. However, it is important to emphasize that such tools are intended to augment, not replace, clinical expertise. The final decision regarding warfarin dosing and monitoring frequency would remain with the clinician, who integrates the XAI-generated insights with their overall assessment of the patient. Future implementation research should explore optimal interface designs, clinician training requirements, and the impact of such tools on clinical outcomes and workflow efficiency.

## 5. Conclusions

This study demonstrated that integrating XAI with ML transforms pharmacogenomic prediction from a statistical exercise into an interpretable clinical tool. While ensemble models like Random Forest and XGBoost showed modest predictive accuracy for poor anticoagulation control with limited specificity for identifying adequately controlled patients, the principal value lies in the XAI-driven insights into model behavior from our dataset. We elucidated that predictions as determined by the model are governed by a core set of genetic (VKORC1, CYP4F2, and CYP2C9) and clinical (SAMe-TT2R2 score, drug interactions) features, and critically, suggesting their distinct mechanistic roles within the model’s decision framework: VKORC1 serves as a stable risk determinant, whereas high-variance CYP2C9 genotypes are the primary drivers of predictive discordance and clinical complexity. The modest discriminative performance underscores that predictive accuracy remains a significant challenge, and these findings should be viewed primarily as demonstrating a methodological approach for generating hypotheses rather than establishing clinically applicable tools. However, given the limitations of our validation approach, including the absence of external validation and cross-validation, these findings should be interpreted as demonstrating a methodological framework rather than establishing a clinically ready predictive tool. The approach enhances transparency and generates hypotheses for future investigation, but substantial additional validation work is required before clinical application can be considered. By revealing the reasons behind both correct and incorrect predictions, this approach enhanced the transparency and trustworthiness of AI in medicine. Although direct clinical application is not yet warranted given the modest performance absence of external validation, this work provides a foundational framework for generating hypotheses and developing future, more accurate models for personalized warfarin management.

## Figures and Tables

**Table 1:** Comparison of characteristics between cohorts.

Variable	Subgroup	Training (n = 163)	Testing (n = 69)	*p* Value
Age	Mean (SD)	65.4 (13.5)	67.5 (13.0)	0.347
CYP2C9	*1/*1	111 (68.1%)	49 (71.0%)	0.657
	*1/*2	31 (19.0%)	11 (15.9%)	
	*1/*3	17 (10.4%)	6 (8.7%)	
	*2/*2	3 (1.8%)	1 (1.4%)	
	*2/*3	1 (0.6%)	1 (1.4%)	
	*3/*3	0 (0.0%)	1 (1.4%)	
CYP4F2	C/C	61 (37.4%)	30 (43.5%)	0.625
	C/T	77 (47.2%)	28 (40.6%)	
	T/T	25 (15.3%)	11 (15.9%)	
VKORC1	C/C	71 (43.6%)	29 (42.0%)	0.519
	C/T	65 (39.9%)	32 (46.4%)	
	T/T	27 (16.6%)	8 (11.6%)	
CYP2C9 metabolizer status	Intermediate	48 (29.4%)	17 (24.6%)	0.593
	Normal	111 (68.1%)	49 (71.0%)	
	Poor	4 (2.5%)	3 (4.3%)	
Gender	Female	75 (46.0%)	33 (47.8%)	0.913
Anticoagulation status	Adequate	64 (39.3%)	27 (39.1%)	1
	Poor	99 (60.7%)	42 (60.9%)	
SAMeTT2R2 score	0	9 (5.5%)	3 (4.3%)	0.868
	1	88 (54.0%)	40 (58.0%)	
	2	61 (37.4%)	23 (33.3%)	
	3	5 (3.1%)	3 (4.3%)	
Presence of interacting drugs		98 (60.1%)	40 (58.0%)	0.874

**Table 2:** Key evaluation metrics.

Model	Random Forest	XGBoost	Logistic Regression
Accuracy	0.67	0.67	0.59
Sensitivity	0.79	0.83	0.95
Specificity	0.48	0.41	0.04
Precision	0.70	0.69	0.61
Recall	0.79	0.83	0.95
F1 Score	0.74	0.75	0.74
AUC [95% CI]	0.67 [0.53, 0.77]	0.68 [0.52, 0.78]	0.50 [0.2, 0.74]
Youden J	0.27	0.24	−0.01
PPV	0.79	0.83	0.95
NPV	0.48	0.41	0.04
PLR	1.52	1.41	0.99
NLR	0.45	0.41	1.29
MAE	0.44	0.40	0.48
RMSE	0.47	0.48	0.50
MSE	0.22	0.23	0.25
Brier score	0.22	0.23	0.25
Log loss	0.62	0.67	0.70
Calibration slope	0.94	0.60	0.059
TP	33	35	40
TN	13	11	1
FP	9	7	2
FN	14	16	26
OR [95% CI]	3.41 [1.2, 9.8]	3.4 [1.1, 10.5]	0.77 [0.07, 8.9]

AUC: Area-under-curve; PPV: Positive predictive value; NPV: Negative predictive value; PLR: Positive likelihood ratio; NLR: Negative likelihood ratio; MAE: Mean absolute error; RMSE: Root mean square error; MSE: Mean square error; TP: True positive; TN: True negative; FP: False positive; and FN: False negative; OR: Odds ratio.

**Table 3:** Comparison of Contribution of Individual Feature Between Concordant and Discordant Cases.

Concordance	Group-Feature	Count	Mean SHAP	SD SHAP	Mean Absolute SHAP	SD Absolute SHAP	Positive Count	Negative Count	Percentage of Positive Counts
Concordant	Genotype-CYP2C9	141	0.91	2.67	2.31	1.61	73	68	51.77
	Genotype-VKORC1	125	1.26	0.86	1.44	0.49	111	14	88.80
	Demographic-Age	73	0.09	1.49	1.42	0.43	44	29	60.27
	Genotype-CYP4F2	166	0.53	1.26	1.26	0.53	102	64	61.45
	Clinical-SAMe-TT2R2 Score	200	0.59	1.29	1.21	0.72	142	58	71.00
	Clinical-Drug Interaction	115	−0.50	0.98	1.08	0.20	41	74	35.65
	Demographic-Gender	155	0.05	1.00	1.00	0.08	75	80	48.39
Discordant	Genotype-CYP2C9	9	2.15	4.75	3.44	3.79	5	4	55.56
	Demographic-Age	11	−0.32	1.52	1.44	0.39	5	6	45.45
	Clinical-SAMe-TT2R2 Score	45	0.73	1.67	1.40	1.15	31	14	68.89
	Genotype-VKORC1	25	0.94	0.95	1.26	0.40	20	5	80.00
	Genotype-CYP4F2	30	0.23	1.26	1.17	0.47	15	15	50.00
	Clinical-Drug Interaction	27	−0.47	1.00	1.07	0.20	10	17	37.04
	Demographic-Gender	33	0.11	1.02	1.00	0.08	17	16	51.52

## Data Availability

The original contributions presented in this study are included in the article. Further inquiries can be directed to the corresponding author(s).
